# Systematic review: occupational sedentary behaviour and common mental health symptoms

**DOI:** 10.1093/occmed/kqaf072

**Published:** 2025-09-22

**Authors:** M Jin, M Swainson, C Wang, A Morris

**Affiliations:** Faculty of Health and Medicine, Lancaster University, Health Innovation One, Sir John Fisher Drive, Lancaster, LA1 4AT, United Kingdom; Faculty of Health and Medicine, Lancaster University, Health Innovation One, Sir John Fisher Drive, Lancaster, LA1 4AT, United Kingdom; Moray House School of Education and Sport, University of Edinburgh, Holyrood Road, Edinburgh, EH8 8AQ, United Kingdom; Faculty of Health and Medicine, Lancaster University, Health Innovation One, Sir John Fisher Drive, Lancaster, LA1 4AT, United Kingdom

## Abstract

**Background:**

Adults accumulate high volumes of daily sedentary behaviour at work, which over time has been associated with negative effects on mental health. Poor mental health, in turn, is associated with increased errors, absenteeism rates, and reduced productivity. Despite this, few studies have examined how occupational sedentary behaviour relates to symptoms of common mental health symptoms.

**Aims:**

To explore the associations between occupational sedentary behaviour and common mental health symptoms.

**Methods:**

A systematic search was conducted following the PRISMA guidelines from CINAHL, Medline, PsycInfo, SPORTDiscus and Web of Science. The JBI checklist was used to assess methodological quality. The best-evidence synthesis was employed to explore associations between occupational sedentary behaviour and mental health symptoms. Synthesis categorized the measurement of mental health symptoms across the selected studies into four groups, including depression, anxiety, combined symptoms of depression and anxiety, and stress.

**Results:**

Five cross-sectional studies from Australia, Sweden, the UK, and the US were included. Overall, mixed results were found, including both positive and no associations between occupational sedentary behaviour and combined common mental health symptoms. The limited evidence currently indicates that there are no independent associations between occupational sedentary behaviour and depression, anxiety and stress.

**Conclusions:**

Current evidence is insufficient to establish a definitive association between occupational sedentary behaviour and common mental health symptoms. However, this review identified important gaps that call for further investigation, specifically into the occupational domain of sedentary behaviour, understanding sedentary behaviour patterns across different occupations and considering job characteristics when exploring its relationship with mental health.

## Teaser

This systematic review aims to explore the association between occupational sedentary behaviour and common mental health symptoms. While this review, synthesizing existing studies, finds insufficient evidence to establish an association, previous research has noted a higher risk of common mental health symptoms associated with prolonged workplace sitting. The findings suggest that further research is needed to explore the mental health effects of occupational sedentary behaviour, in order to inform the development of targeted workplace interventions.

## INTRODUCTION

The prevalence of common mental health disorders such as depression, anxiety and stress among working-age adults poses a critical public health concern, which significantly impacts individual well-being and productivity [1]. In addition, poor mental health can adversely affect individuals’ work performance, resulting in reduced pace, increased errors and elevated absenteeism [2]. Globally, approximately 1 billion individuals suffer from mental health disorders, and it is estimated that the global economy incurs an annual loss of $1 trillion as a direct result of reduced productivity stemming from common mental health disorders, specifically depression and anxiety [3]. Consequently, understanding the factors influencing mental health disorders is of significance to public health.

Daily sedentary behaviour in adults has been shown to be deleteriously associated with common mental health disorders with the risk of depression increasing by 5% for each hour accumulated of daily television watching [4]. It is estimated that adults spend about 8.2 h/day (ranging from 4.9 to 11.9 h/day) being sedentary [5]. This might expose adults to a high risk of negative mental health outcomes.

Although previous research has consistently demonstrated a negative association between daily sedentary behaviour and mental health, recent studies have suggested a more nuanced perspective, indicating that not all forms of sedentary behaviour are linked to adverse mental health outcomes. Based on the social-ecological model of sedentary behaviour, there are different domains that have been identified, which include leisure and occupational sedentary behaviour [6]. Current evidence predominantly shows positive associations between leisure­related sedentary behaviour and mental disorders, such as watching TV [4]. However, there is evidence suggesting that office work–related sedentary behaviour is linked to lower hazards of mental disorders [7].

Key learning points **What is already known about this subject:**

- Prolonged sedentary behaviour has been found to be associated with negative mental health outcomes;
  However, this conclusion is largely based on total daily sitting time, and few studies have explored the mental health effects of specific domains of sedentary behaviour.

**What this study adds:**

- This review adopted a non-monolithic understanding of the concept of sedentary behaviour and found that existing evidence is insufficient to establish an association between occupational sedentary behaviour and mental health.

**What impact this may have on practice or policy:**

- The findings consolidate the necessity of focusing on specific domains of sedentary behaviour.
- Considering that sedentary behaviour is characterized by various factors, such as nature of job or creativity of the tasks, more meticulous classification and investigation are needed; these efforts will inform the development of targeted workplace health promotion interventions.

Considering that the workplace is an important setting where high volumes of daily sedentary behaviour are accumulated [8], and depending on the job role, desk-based work accounts for 60–90% of an individual’s daily sitting time [9]. It is essential to determine whether there is an association between occupational sedentary behaviour and common mental health symptoms. Therefore, the aim of this review was to explore the potential associations between occupational sedentary behaviour and common mental health symptoms, including depression, anxiety and stress.

## METHODS

A systematic review was undertaken following the Preferred Reporting Items for Systematic Reviews and Meta-Analyses (PRISMA) framework [10]. The protocol was registered with PROSPERO (registration number: CRD42024517946).

An initial systematic literature search was conducted in April 2023, and a further search was conducted in January 2024 to check for additional studies. The following databases were used: PsycINFO, CINAHL, MEDLINE Complete, SPORTDiscus and Web of Science. The selection of databases was based on previous studies and advice from Lancaster University librarians. There were no restrictions on publication dates and language.

The key terms used were ‘sedentary behaviour’, ‘work’, and ‘mental health’. The MeSH terms (Medical Subject Headings) were used. Full search strings are included in the Supplementary Document S1 (available as Supplementary data at *Occupational Medicine* Online).

The study eligibility criteria are as follows: (i) the study included working-age adults (≥18 years old) who were employed in desk-based jobs (in person, not remote); (ii) participants had no chronic physical conditions, e.g. cancer or diabetes; (iii) any measurement of occupational sedentary behaviour was included, such as self-reported logs, questionnaires, standardized scales, pedometer and/or accelerometer device–based measurements; (iv) any measurement of mental health was included, such as standardized psychological scales, questionnaires and clinical diagnoses of mental health disorders; (v) the study design included observational studies or experimental studies, such as cross-­sectional studies, cohort (longitudinal) studies and randomized or non-randomized controlled trial interventions; and (vi) intervention studies focused on the direct association between sedentary behaviour and mental health. Exclusion criteria are as ­follows: (i) papers written in languages other than English; (ii) no measurement or report of germane mental health issues (i.e. measuring well-being rather than depression, anxiety and stress); (iii) leisure or non-occupational sedentary behaviour; (iv) work from home; (v) study protocols; (vi) child, adolescent, or older adult participants; (vii) intervention studies that primarily aimed at promoting physical activity; and (viii) studies reported the effects of an intervention on either mental health or sedentary behaviour, but not their association.

Retrieved papers were initially input into EndNote for deduplication. All papers were then uploaded into the online systematic review tool, Rayyan (https://www.rayyan.ai/), for screening. The first and third authors independently conducted the screening process, including title, abstract and full text. Disagreements were resolved by discussion.

A customized data extraction form was developed and pretested by the primary author. Key elements extracted included general study information (authors, publication time, country) and methodological characteristics including the study design, participant characteristics (sample size, age, sex), occupational sedentary behaviour and its measurement, indicators and measurements of mental health and outcomes on the association between occupational sedentary behaviour and mental health.

The Joanna Briggs Institute (JBI) checklist for cross-sectional studies [11] was utilized for the methodological quality evaluation. It comprises eight items that assess the included studies based on sample selection, the validity and reliability of measurement, confounding factors and statistical analysis. This review adopted cut-offs from previous research while adhering to the JBI checklist authors’ recommendation by presenting the results of the critical appraisal in a tabulated format for each question [12, 13]. To assess the risk of bias, the studies’ scores were categorized into three levels: a low risk of bias for studies with 70% or more of the items scored ‘Yes’; a moderate risk for those with 50%–69% ‘Yes’ scores; and a high risk for studies scoring below 50% ‘Yes’.

This systematic review investigated outcomes identifying a direct (statistical) association between occupational sedentary behaviour and mental health symptoms, including depression, anxiety and stress.

This review used a best-evidence synthesis approach [14] to investigate the association between occupational sedentary behaviour and mental health. This is an alternative to meta-­analysis and traditional narrative review [14], aiming to incorporate the ‘best evidence’ available (i.e. studies of the highest quality) to comprehensively analyse the included literature. The rationale for adopting this approach stemmed from the limited number of studies included and the heterogeneity in measures of effect across findings (i.e. odds ratio, risk ratio, correlation and prevalence), which made quantitative meta-analysis unsuitable. Meanwhile, the traditional narrative synthesis might face challenges of lacking transparency and replicability [15]. The best-evidence method, however, has been widely used in previous systematic reviews examining the association between sedentary behaviour and health outcomes [16–18].

In this study, three levels of evidence strength were utilized. Strong evidence is defined as consistent findings derived from two or more high-quality studies. Moderate evidence encompasses two scenarios: either consistent results observed in one high-quality study alongside at least one lower quality study or consistent findings observed across two or more lower quality studies. Finally, insufficient evidence indicates either the availability of only one study or inconsistent results reported in two or more studies.

Consistent findings referred to at least 75% of the studies showing results in the same direction [18]. Studies with weak quality were disregarded in the evidence synthesis if two or more studies were of strong or moderate methodological quality [17].

## RESULTS

**Figure 1.**
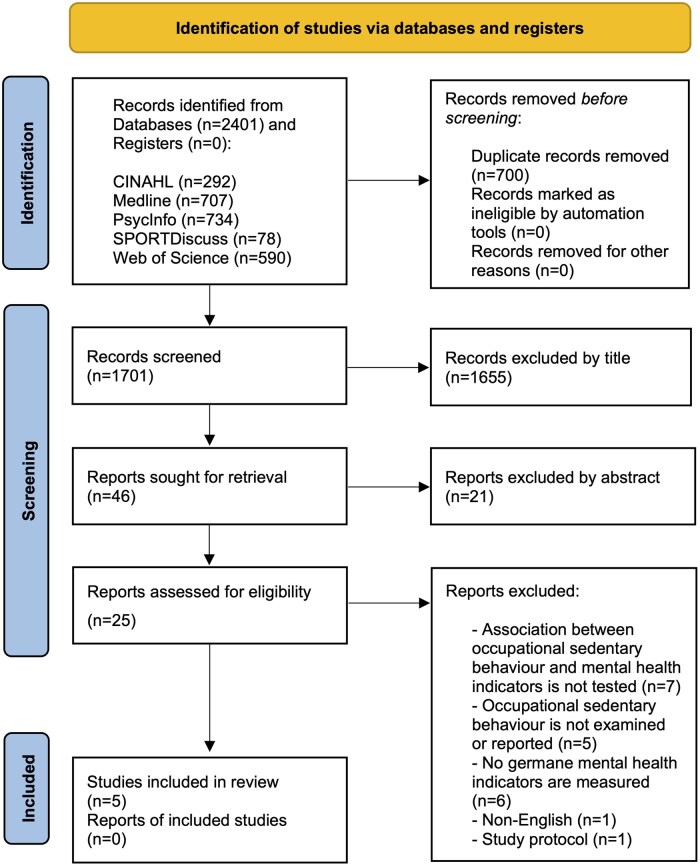
The PRISMA flowchart of the study.

The study selection procedure involved five steps, as illustrated in the PRISMA flow diagram (Figure 1). Of the 2401 identified records, five studies met the eligibility criteria and were included in the review. The majority of studies were excluded during the title (*n* = 1655) and abstract (*n* = 21) screening phase primarily for the following reasons: (i) focused on populations other than working-age adults, (ii) focused on physical activity, and (iii) focused on leisure sedentary behaviour. During the full-text screening stage, an additional 20 studies were excluded. Details of the procedure and exclusion reasons are shown in Figure 1.

The five studies involved a total of 29045 participants (ranging from 77 to 23644). Contributions were published between 2013 and 2021, originating from Australia (*n* = 2) [19, 20], Sweden (*n* = 1) [21], UK (*n* = 1) [22], and USA (*n* = 1) [23]. All five articles were cross-sectional designs, with no longitudinal, interventional or experimental studies meeting the inclusion criteria. Detailed descriptions of each study are provided in Table 1.

**Table 1.** Studies investigating the association between occupational sedentary behaviour and common mental health disorders (i.e. depression, anxiety and stress)

Study	Study design and sample	OSB and measurement	MH indicator(s) and measurement	Outcomes
Kilpatrick *et al.* (2013) Australia	Cross-sectional study *n* = 3367 Age = 47 72% Female	**Indicator:** Sitting at work **Self-report measures:** First estimate time spent at the workplace, then estimate time spent sitting at the workplace.	**Components:** A state of emotional suffering characterized by symptoms of depression and anxiety **Self-report measures:** 10-item Kessler Psychological Distress Scale	**In adjusted model:** **Men** Moderate distress = + High distress = 0 Very high = 0 **Women** Moderate distress = + High distress = + Very high = 0
Rebar *et al.* (2014) Australia	Cross-sectional study *n* = 1843 Age = 58 55% Female	**Indicator:** Work sitting **Self-report measures:** 10-item Workforce Sitting Questionnaire	**Components:** Symptoms of depression, anxiety, and stress **Self-report measures:** 21-item Depression, Anxiety, and Stress Scale	Depression = 0 Anxiety = 0 Stress = 0
Ryde *et al.* (2019) The UK	Cross-sectional study *n* = 77 Age = 40.8 78% Female	**Indicator:** Occupational sedentary behaviour **Device-based measures:** Sitting pad	**Components:** Stress **Biomarker:** Hair cortisol **Self-report measures:** 10-item Cohen Perceived Stress Scale	**Biomarker **= 0 **Self-report stress **= 0
Hallgren *et al.* (2020) Sweden	Cross-sectional study *n* = 23644 Age = 42 57% Female	**Indicator:** Occupational sedentary behaviour **Self-report measures:** Assessed with the question ‘I sit still at work…’ with five proportion responses.	**Components:** Symptoms of depression and anxiety **Self-report measures:** Assessed with the question ‘I experience worry, depressed mood or anxiety…’ with five frequency responses.	**In adjusted model:** Almost always = + 75% of time = 0 50% of time = 0 25% of time = 0 Almost never = 0
Gallagher *et al.* (2021) The USA	Cross-sectional study *n* = 114 Age = 39 74.5% Female	**Indicator:** Occupational sedentary behaviour **Device-based measures:** Accelerometer	**Components:** Stress **Self-report measures:** Ecological Momentary Assessment (assess stress by a single item Likert scale from 1 to 10).	Average stress = 0

*Note.* OSB = occupational sedentary behaviour, MH = mental health; ‘+’ = occupational sedentary behaviour is associated with worse mental health conditions/higher risk of mental health issues, ‘0’ = no association is found or reported.

Occupational sedentary behaviours were assessed using both self-reported and device-based measurements across the included articles. Three studies utilized self-reported measurements, which included estimations of sitting time [20, 21] and validated questionnaires, i.e. the Workforce Sitting Questionnaire [19]. Two studies employed device-based measurements, including sitting pads [22] and accelerometers, ActivPAL3 [23].

Mental health indicators were evaluated using self-reported measurements and biological indicators across the five articles. Two of the five studies examined the combined symptoms of depression and anxiety, including one study assessed by asking participants to rate their mental experience on a five-point scale [21], while the other used a standardized Kessler Psychological Distress scale [20]. Another two of the five studies focused solely on assessing stress, with one utilizing both a biological indicator (Hair Cortisol) and a standardized stress scale (Cohen Self­Perceived Stress Scale) [22]; one employing a self-reported ecological momentary assessment [23]. The remaining study assessed all symptoms of depression, anxiety and stress individually using a standardized scale, i.e. Depression, Anxiety Stress Scale [19].

Confounding variables were identified and measured by questionnaire or scale, including sex and gender [19–23], age [19–23], ethnic background [22, 23], income [19, 22, 23], education [19–21, 23], smoking status [21], marital or relationship status [20, 23], physical functioning [20], weight status [20], BMI [20, 21, 23], pain [21], presence of chronic conditions [19], self-reported perceived health [22], exercise frequency [21], light physical activity [20], moderate-to-vigorous physical activity [22], effort–reward imbalance (work-related stress) [20], job level [19], employment status and work condition [20, 22], work category [20], average workday length [22], hours worked in the last 7 days [22], and qualification [22].

Overall, the included articles showed a moderate to low risk of bias. Four studies were rated as low risk of bias and one study was rated as moderate. Rating details of each article are presented in Table 2.

**Table 2.** JBI Checklist for analytical cross-sectional studies [11]

Study	Q1	Q2	Q3	Q4	Q5	Q6	Q7	Q8	%Yes	Risk of bias	Quality
Kilpatrick *et al.* (2013)	N	Y	N	Y	Y	Y	Y	Y	75%	Low	High
Rebar *et al.* (2014)	N	Y	Y	Y	Y	Y	Y	Y	87.5%	Low	High
Ryde *et al.* (2019)	Y	Y	Y	Y	Y	Y	Y	Y	100%	Low	High
Hallgren *et al.* (2020)	N	Y	N	N	Y	Y	N	Y	50%	Moderate	Moderate
Gallagher *et al.* 2021	Y	Y	Y	Y	Y	Y	Y	N	87.5%	Low	High

*Note.* The degree of bias risk was assessed as high when the study obtained up to 49% of affirmative responses, moderate when the study obtained responses ranging from 50% to 69%, and low when the study obtained more than 70% of affirmative responses. The symbols ‘Y’, ‘N’, ‘?’ and ‘N/A’, respectively, represent the affirmative, negative, unclear and not applicable response.

Of the five studies, three high-quality studies (60%) found null associations between occupational sedentary behaviour and mental health components [19, 22, 23]. One high-quality study (20%) found positive associations between the two variables [20], and one moderate-quality study (20%) found mixed findings (i.e. positive and null) [21].

Based on the best evidence synthesis, there was insufficient evidence to determine the association between occupational sedentary behaviour and common mental health symptoms, due to the mixed results (i.e. positive and null associations) across the included studies. Results and supporting articles are summarized in Table 3.

**Table 3.** Synthesis results and supporting evidence

Synthesis groups and results	Supporting article	
Combined symptoms of depression and anxiety: Positive and no association (insufficient evidence)	**×**	(Kilpatrick *et al.*, 2013)
		(Rebar *et al.*, 2014)
		(Ryde *et al.*, 2019)
	**×**	(Hallgren *et al.*, 2020)
		(Gallagher *et al.*, 2021)
Depression: No association (insufficient evidence)		(Kilpatrick *et al.*, 2013)
	**×**	(Rebar *et al.*, 2014)
		(Ryde *et al.*, 2019)
		(Hallgren *et al.*, 2020)
		(Gallagher *et al.*, 2021)
Anxiety: No association (insufficient evidence)		(Kilpatrick *et al.*, 2013)
	**×**	(Rebar *et al.*, 2014)
		(Ryde *et al.*, 2019)
		(Hallgren *et al.*, 2020)
		(Gallagher *et al.*, 2021)
Stress: No association (strong evidence)		(Kilpatrick *et al.*, 2013)
	**×**	(Rebar *et al.*, 2014)
	**×**	(Ryde *et al.*, 2019)
		(Hallgren *et al.*, 2020)
	**×**	(Gallagher *et al.*, 2021)

Regarding combined symptoms of depression and anxiety, a high-quality study demonstrated positive associations, whereas a moderate-quality study reported mixed results (positive and null associations). Specifically, one high-quality study investigated the association between work sedentary behaviour and psychological distress among employees [20]. The study found that men who sit for more than 6 h a day show a higher prevalence of moderate psychological distress compared to those who sit for less than 3 h a day. Similarly, women sitting for more than 6 h a day experience a higher prevalence of both moderate and high psychological distress. One moderate-quality study found mixed results in examining the association between occupational sedentary behaviour and the frequency of combined depression and anxiety [21]. Among the five levels of sedentary behaviour amount (almost never, 25% of time, 50% of time, 75% of time and almost always), no associations were found except for the level ‘almost always’.

Regarding symptoms of depression and anxiety individually, one high-quality study using a standardized workforce sedentary behaviour scale and a mental health scale found no association with either condition [19].

Regarding stress, three high-quality studies found no associations. Specifically, one study used a standardized workforce sedentary behaviour scale and a mental health scale to explore the association, and no significant result was found [19]. One study used device-based measurement to capture occupational sedentary behaviour and self-reported stress and found no association [23]. The final one used objective measurement of both occupational sedentary behaviour and stress, and no association was found [22].

## DISCUSSION

From an occupational domain-centred perspective, this review found insufficient evidence to establish an association between occupational sedentary behaviour and common mental health symptoms. Specifically, for combined symptoms of depression and anxiety, mixed results were found, including positive and no associations. For depression, anxiety and stress, individually, insufficient evidence indicates an association. However, with only five studies published specifically focusing on the work environment, it is clear that evidence is scarce in this area of research. To the authors’ knowledge, this is the first systematic review to synthesize the evidence of associations between occupational sedentary behaviour and common mental health symptoms.

This review’s insufficient evidence regarding an association between depression or anxiety and occupational sedentary behaviour contrasts with prior systematic reviews that have demonstrated total sedentary behaviour to be associated with an increased risk of these symptoms [24–26].

The primary factor contributing to this discrepancy is the scarcity of studies specifically focusing on sedentary behaviour within occupational contexts. This focus is crucial, however, given that a significant proportion of modern employment is predominantly sedentary with low physical demands (e.g. office work, vehicle operation, call centres). Although some autonomy regarding movement may exist, opportunities for physical activity during work hours are often constrained by the inherent nature of the work [27]. Investigating the proportion of time spent sedentary during work and whether this differs from non-working hours can help inform workplace health and well-being strategies. Understanding sedentary patterns across diverse occupations is also beneficial for tailored intervention development, as occupations exhibit different regularities. For example, call-centre employees exhibited longer sedentary bouts than office workers [28].

Second, the nature of occupational sedentary behaviour may offer some protection for mental health, which could explain the discrepancy. This is because occupational sedentary behaviour inherently involves greater cognitive engagement, encompassing tasks that require working memory and logical reasoning. Cognitive engagement is associated with better mental health [29], and is a major component of ‘mentally active sedentary behaviour’ [30]. A recent meta-analysis suggests that ‘mentally active sedentary behaviour’ is not associated with depression risk [30], a finding consistent with the null association observed in this review. Depression and anxiety are common comorbid mood disorders [31], and research shows that both symptoms exhibit similar responses to risk and protective factors [32]. This may explain why total sedentary behaviour is associated with an increased risk of both conditions, while cognitively engaging occupational sedentary behaviour does not.

Moreover, understanding the job characteristics is crucial when exploring the relationship between occupational sedentary behaviour and mental health. While occupational sedentary behaviour generally involves cognitive engagement, the varying levels of mental activity across occupations may have different impacts on mental health. Repetitive tasks in some job roles could be detrimental for mental health [33], such as assembly line. Future research should focus on specific occupations to identify their distinct characteristics that influence workplace behaviour and mental health outcomes. Meanwhile, employers are expected to take responsibility for preventing or managing these outcomes. For instance, the Health and Safety Executive advises stress risk assessments to help resolve related issues [34], whether stemming from overwork or boredom.

Regarding stress, all included studies found no association with occupational sedentary behaviour, aligning with a previous review on total sedentary behaviour that found insufficient evidence [17]. However, current findings should be interpreted cautiously due to the limited number of studies. Unlike depression and anxiety, which are chronic mental disorders, stress is an immediate response to external pressures [35]. Given this, a stronger association with occupational sedentary behaviour was anticipated, but none was found in this review. It is possible that unmeasured workplace stressors, such as job demands and workloads [36], may diminish sedentary behaviour’s influence on stress. Therefore, further investigation is needed to explore the complex interplay between occupational sedentary behaviour, workplace stressors and stress, especially since unmanaged stress can escalate into chronic mental health disorders [37].

The included studies examined several confounding variables that could mediate or moderate the relationship between total sedentary behaviour and mental health. Sex and gender are important factors; one study showed sex contributes to depression risk but not anxiety [19], while another found differential responses to occupational sedentary behaviour between males and females [20]. Additionally, physical activity, known to benefit mental health, was found in three studies to attenuate certain effects of sedentary behaviour [19, 23]. The cause and effect between occupational sedentary behaviour and mental health is challenging to define because it is multifaceted, dynamic and potentially bidirectional [38]. Nevertheless, despite this complexity, current World Health Organization (WHO) guidelines emphasize that reducing sedentary behaviour is important for health [39].

While this review followed rigorous, replicable methods, its findings should be interpreted cautiously due to limitations in the evidence. First, with only five studies included, the conclusions are inherently limited in generalizability and should be viewed as preliminary. However, the small sample size reflects the early stage of research into the nuanced impact of sedentary behaviour on mental health. Second, all included studies were cross-sectional, preventing causal inferences between occupational sedentary behaviour and mental health. Nonetheless, attention was primarily given to sedentary behaviour’s potential influence on mental health, as it is modifiable and aligns with public health recommendations [39]. Another limitation of this review is the heterogeneity in how occupational sedentary behaviour was measured across studies. This warrants cautious interpretation but also highlights the need for future reviews to include more consistent and objective measures, which aligns with the WHO’s recommendation to incorporate device-based measurements [40].

In conclusion, this review examined existing cross-sectional literature on the association between occupational sedentary behaviour and common mental health symptoms. Although insufficient evidence was found to establish clear associations, the scarcity of research highlights several gaps for future studies, including (i) investigate the specific domain of occupational sedentary behaviour, (ii) use device-based measurements to understand sedentary behaviour patterns across different occupations and (iii) understand how job characteristics influence the relationship between occupational sedentary behaviour and mental health. These efforts will contribute to developing targeted workplace interventions for reducing sedentary behaviour and promoting mental health.

## Supplementary Material

kqaf072_Supplementary_Data
