# Supplementary material for: Systematic review: occupational sedentary behaviour and common mental health symptoms
Source: Occup Med (Lond). 2025 Sep 22;75(6):275–81. doi: 10.1093/occmed/kqaf072 (PMC12449258; doi:10.1093/occmed/kqaf072)
Supplement: kqaf072_Supplementary_Data [file kqaf072_supplementary_data.docx]

**This supplementary material is the full search strategy for each database.**

**CINAHL (n=292)**

S1 ( (MH "Life Style, Sedentary+") OR (MH "Screen Time") ) OR TI ( ((sedentary OR seated OR sitting) n3 (behav* OR time)) OR desk-based ) OR AB ( ((sedentary OR seated OR sitting) n3 (behav* OR time)) OR desk-based )

S2 ( (MH "Work+") OR (MH "Stress, Occupational+") ) OR TI ( employe* OR workplace OR occupation* OR context OR ((office) n3 (work*)) ) OR AB ( employe* OR workplace OR occupation* OR context OR ((office) n3 (work*)) )

S3 ( (MH "Mental Health") OR (MH "Depression+") OR (MH "Anxiety+") OR (MH "Stress+") OR (MH "Stress, Occupational+") ) OR TI ( ((mental OR Psyc*) n3 health) OR depress* OR anxiety OR stress ) OR AB ( ((mental OR Psyc*) n3 health) OR depress* OR anxiety OR stress )

S1 AND S2 AND S3

**Medline Complete (n=707)**

S1 (MH "Sedentary Behavior") OR TI ( ((sedentary OR seated OR sitting) n3 (behav* OR time)) OR desk-based ) OR AB ( ((sedentary OR seated OR sitting) n3 (behav* OR time)) OR desk-based )

S2 ( (MH "Work+") OR (MH "Workplace+") ) OR TI ( employe* OR workplace OR occupation* OR context OR ((office) n3 (work*)) ) OR AB ( employe* OR workplace OR occupation* OR context OR ((office) n3 (work*)) )

S3 ( (MH "Population Health") OR (MH "Mental Health") OR (MH "Occupational Health") ) OR TI ( ((mental OR Psyc*) n3 health) OR depress* OR anxiety OR stress ) OR AB ( ((mental OR Psyc*) n3 health) OR depress* OR anxiety OR stress )

S1 AND S2 AND S3

**APA PsycInfo (n=734)**

S1 ( DE "Sedentary Behavior" OR DE "Computer Usage" OR DE "Computer Searching" OR DE "Internet Usage" OR DE "Online Behavior" OR DE "Screen Time" OR DE "Smartphone Use" OR DE "Computers" OR DE "Screen Time" ) OR TI ( ((sedentary OR seated OR sitting) n3 (behav* OR time)) OR desk-based ) OR AB ( ((sedentary OR seated OR sitting) n3 (behav* OR time)) OR desk-based )

S2 ( DE "Occupations" OR DE "Employee Well Being" OR DE "Occupational Stress" OR DE "Personnel" OR DE "Workplace Intervention" ) OR TI ( employe* OR workplace OR occupation* OR context OR ((office) n3 (work*)) ) OR AB ( employe* OR workplace OR occupation* OR context OR ((office) n3 (work*)) )

S3 ( DE "Mental Health" OR DE "Occupational Stress" OR DE "Occupational Health Psychology" OR DE "Occupational Health" OR DE "Depression (Emotion)" OR DE "Stress" OR DE "Occupational Stress" OR DE "Anxiety" ) OR TI ( ((mental OR Psyc*) n3 health) OR depress* OR anxiety OR stress ) OR AB ( ((mental OR Psyc*) n3 health) OR depress* OR anxiety OR stress )

S1 AND S2 AND S3

**SPORTDiscus (n=78)**

S1 ( DE "SEDENTARY behavior" OR DE "SEDENTARY lifestyles" OR DE "SEDENTARY people" ) OR TI ( ((sedentary OR seated OR sitting) n3 (behav* OR time)) OR desk-based ) OR AB ( ((sedentary OR seated OR sitting) n3 (behav* OR time)) OR desk-based )

S2 ( DE "OCCUPATIONAL diseases" OR DE "OCCUPATIONAL health services" ) OR TI ( employe* OR workplace OR occupation* OR context OR ((office) n3 (work*)) ) OR AB ( employe* OR workplace OR occupation* OR context OR ((office) n3 (work*)) )

S3 ( DE "PUBLIC health" OR DE "PSYCHOLOGICAL stress" OR DE "MENTAL illness" OR DE "MENTAL health" OR DE "MENTAL depression" OR DE "PSYCHOLOGICAL stress" OR DE "ANXIETY" ) OR TI ( ((mental OR Psyc*) n3 health) OR depress* OR anxiety OR stress ) OR AB ( ((mental OR Psyc*) n3 health) OR depress* OR anxiety OR stress )

S1 AND S2 AND S3

**Web of Science (590)**

Topic (((sedentary OR seated OR sitting) near/3 (behav* OR time)) OR desk-based) AND Topic (employe* OR workplace OR occupation* OR context OR ((office) near/3 (work*))) AND Topic (((mental OR Psyc*) near/3 health) OR depress* OR anxiety OR stress)
